# Improving hospital-based opioid substitution therapy (iHOST): protocol for a mixed-methods evaluation

**DOI:** 10.3310/nihropenres.13534.1

**Published:** 2024-03-13

**Authors:** Dan Lewer, Michael Brown, Adam Burns, Niamh Eastwood, Rosalind Gittins, Adam Holland, Vivian Hope, Aubrey Ko, Penny Lewthwaite, Ann-Marie Morris, Adrian Noctor, Andrew Preston, Jenny Scott, Erica Smith, Sedona Sweeney, Nerissa Tilouche, Marisha Wickremsinhe, Magdalena Harris

**Affiliations:** 1 Bradford Institute for Health Research, Bradford Teaching Hospitals NHS Foundation Trust, Bradford, England, BD9 6RJ, UK; 2 University College London, London, England, WC1E 6AE, UK; 3 University College London Hospitals NHS Foundation Trust, London, NW1 2PG, UK; 4 Exchange Supplies, Dorchester, DT1 1RD, UK; 5 Leeds Teaching Hospitals NHS Trust, Leeds, LS9 7TF, UK; 6 Release, London, E1 8AN, UK; 7 Aston University, Birmingham, England, B4 7ET, UK; 8 University of Bristol, Bristol, England, BS8 1QU, UK; 9 Liverpool John Moores University, Liverpool, England, L2 2QP, UK; 10 London School of Hygiene and Tropical Medicine, London, WC1E 7HT, UK; 11 University Hospital of the North Midlands NHS Trust, Stoke-on-Trent, ST4 6QG, UK

**Keywords:** Opiate Substitution Treatment, Methadone, Buprenorphine, Substance-Related Disorders, Opioid-Related Disorders, Heroin Dependence, Hospitals, Staff Development

## Abstract

**Background:**

Opioid substitution therapy is associated with improved health and social outcomes for people who use heroin and other illicit opioids. It is typically managed in the community and is not always continued when people are admitted to hospital. This causes opioid withdrawal, discharge against medical advice, and increased costs. We are establishing a project called iHOST (improving hospital opioid substitution therapy) to address these problems. This is an applied health research project in which we will develop and evaluate an intervention that aims to improve opioid substitution therapy in three acute hospitals in England. The intervention was developed in collaboration with stakeholders including people who use opioids, hospital staff, and other professionals who work with this group. It includes five components: (1) a card that patients can use to help hospital clinicians confirm their opioid substitution therapy, (2) a helpline for patients and staff, (3) an online training module for staff, (4) a clinical guideline for managing opioid withdrawal in hospital, and (5) ‘champion’ roles at each hospital.

**Methods:**

We will do a mixed-methods study including a quasi-experimental quantitative study and a qualitative process evaluation. The primary outcomes for the quantitative study are discharge against medical advice and emergency readmission within 28 days. We will do a difference-in-difference analysis comparing changes in these outcomes for patients at iHOST sites with changes for patients at control hospitals. The process evaluation will use in-depth interviews, focus groups, and site observations with people who use opioids and staff. We will assess acceptability of the intervention, barriers and facilitators to implementation, and contextual factors impacting outcomes.

**Impact:**

We anticipate that iHOST will improve care for hospital patients who use illicit opioids and/or are receiving community-based opioid substitution therapy. Depending on the results, we will promote the intervention at hospitals across the UK. Dissemination, including through publication, will inform hospital-based services for people who use drugs both in the UK and other countries.

## Plain English Summary

People who use heroin and other illegal opioids can be supported with medically prescribed opioids. These legal medicines, like methadone and buprenorphine, can reduce the need for illegal opioids and improve overall wellbeing. Normally, these treatments are managed in community clinics. They can be provided at stable doses for long periods, sometimes decades.

However, when people are admitted to hospital for treatment of medical problems, they don’t always continue receiving these crucial treatments. This happens for various reasons – sometimes hospital staff do not fully understand how the medicines work, or the hospital might have overly strict rules that restrict their use. As a result, patients can experience opioid withdrawal and pain, which can lead them to leave hospital to buy illegal opioids. It might also cause arguments between staff and patients. Hospitals often focus too much on potential risks associated with these medicines, and sometimes forget the needs of patients who depend on them.

We are addressing these issues in three hospitals in England. Our project consists of five components: (1) a patient card with information about their opioid treatment to speed up prescriptions; (2) a helpline for patients and hospital staff to seek support; (3) an online training module for hospital staff; (4) clinical guidelines for managing opioid withdrawal in hospitals; (5) named ‘champions’ in each hospital to raise the profile of these resources. These components have been developed together with people who use heroin and hospital staff.

We will study the effect of the project using a combination of quantitative and qualitative methods. The quantitative aspect will assess how the project affects the number of patients leaving against medical advice or returning to hospital in emergencies. The qualitative aspect will involve interviews and focus groups to understand how patients and staff perceive the project.

## Introduction

People who use illicit opioids such as heroin have a high rate of hospital admission, particularly due to injecting-related and respiratory infections, exacerbations of chronic respiratory diseases, and gastrointestinal problems related to hepatitis and comorbid alcohol dependence ^1– 3^. In the UK the population using illicit opioids is ageing due a peak in initiation of heroin use in the 1980s and 1990s ^4, 5^. Management and acute treatment of long-term conditions is therefore becoming a more important issue in this population.

Many people who use illicit opioids report poor experiences when admitted to hospital. Qualitative research has found evidence of stigma among hospital staff, diagnostic overshadowing (in which symptoms are attributed to drug use and not investigated to the same extent as other patients), poor pain relief, and limited availability of opioid substitution therapy (OST) ^6– 10^. OST is an intervention that includes prescription of opioids such as methadone or buprenorphine to alleviate opioid withdrawal, reduce illicit drug use, and help provide stability ^11^. In the UK, it is typically managed in community-based clinics. Research in the UK and internationally shows that lack of OST in hospitals is a key barrier to hospital care and means that people are reluctant to go to hospital until symptoms are severe, or may leave hospital against medical advice to access illicit opioids ^8, 12, 13^.

Outcomes for this patient group are poor. For example, approximately 10% of inpatients with a history of using illicit opioids leave against medical advice (compared to approximately 1% of all patients) ^14– 16^; 80% of admissions are unplanned, compared to 50% for all hospital inpatients of the same age ^17^); and the risk of fatal opioid overdose is four times greater in the two weeks after hospital discharge than at other times ^14^.

Various initiatives have been established to improve OST and care for hospital patients who use illicit drugs ^18^. These include addiction liaison teams, in which multidisciplinary specialists support ward staff to care for patients who use drugs and alcohol; bridge clinics, which aim to link patients with community addiction services and provide addiction treatment while these linkages are being made; and training for general medical staff ^18, 19^. These initiatives have mostly been studied in North America. The ‘Improving Hospital Opioid Substitution Treatment’ (iHOST) project is working with three acute hospitals in England to develop, test, and evaluate an intervention to improve opioid withdrawal management and OST provision in acute NHS Hospitals.

## Protocol

### Patient and Public Involvement

The project stemmed from community-identified need, and intervention components were co-produced with people who use heroin and/or receive OST. Our advisory board members, peer researchers, and investigators also include people with past and current experience of opioid use to ensure community accountability. Some of authors listed on the present paper have past or current experience of opioid use.

In quarterly workshops throughout the project we will work with a group of ‘peer experts’ (people who currently use opioids or are on OST) to discuss project progress and findings and iteratively co-create a cultural safety framework to inform hospital care for people who use opioids in England. This participatory approach is core to the iHOST project.

Cultural safety principles focus on the way dominant cultural expectations of healthcare can be experienced as unsafe by marginalised groups ^20– 23^. Cultural safety principles aim to reduce health care practices that cause marginalised patients to feel unsafe and powerless. Developed by nurse academics working with Maori patients in New Zealand, this approach has been translated widely, including for practitioners working with people who use drugs in North America.

### Aims and objectives

iHOST aims to improve OST in hospital settings to reduce delayed presentation, improve care, reduce discharges against medication advice, and reduce emergency readmissions. The objectives are to: (1) optimise iHOST components and test feasibility in a London hospital and associated local drug services; (2) evaluate intervention acceptability, fidelity, reach, costs, and impact; (3) develop and disseminate toolkits for national implementation.

### The intervention

**Figure 1.**
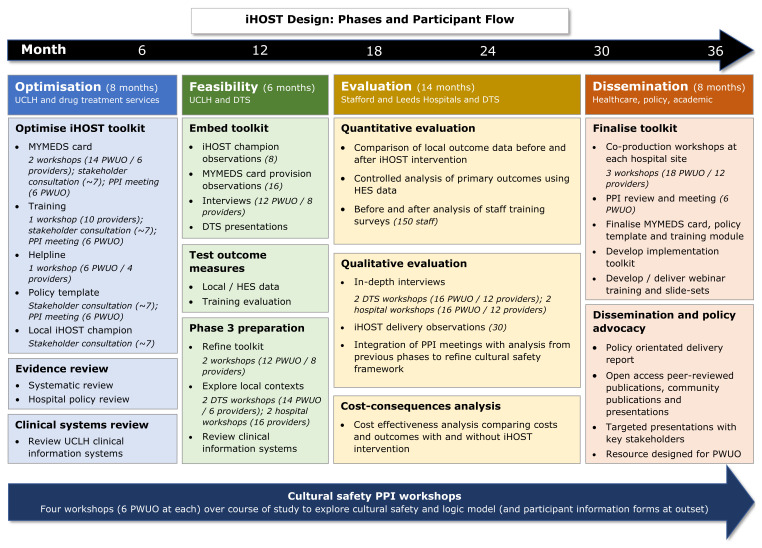
Programme phases.

We will develop and evaluate an intervention (iHOST) to improve OST in acute hospital settings. iHOST will be implemented at three acute hospitals in England: University College London Hospital, St. James University Hospital Leeds, and Royal Stoke University Hospital. The project will be developed in partnership with local drug treatment services. The intervention primarily aims to improve continuation of OST from community to hospital settings but will also inform OST initiation and discharge planning for patients who were not already in receipt of community-based OST. The programme phases are shown in Figure 1, and launch dates are currently anticipated to be:

- University College London Hospital: November 2022 (already launched at the time of publication)
- St James University Hospital Leeds: January 2024
- Royal Stoke University Hospital: January 2024

iHOST is designed to be pragmatic and work in real-world hospitals and drug treatment services. It consists of five components, which are listed below. More information is available at https://www.lshtm.ac.uk/research/centres-projects-groups/ihost.

a) My Meds Card: A patient advocacy card designed for hospital patients who receive OST in the community. This includes information about the importance of timely OST on hospital admission, a space for prescriber and community pharmacy contact details to enable verification of the patient’s OST dose, and an advocacy helpline number (see below). Patients can give the card to hospital clinicians to facilitate OST. The card was co-produced in workshops with people who use opioids and refined in collaboration with hospital clinicians, pharmacy, and drug treatment service providers.
b) A helpline for patients and hospital staff run by the drugs advocacy charity Release ( https://www.release.org.uk/). The helpline supports patients in advocating for OST access and supports hospital staff who have questions about OST.
c) An online training module for hospital staff working in acute admissions and key specialties such as infection control and hepatology. The module aims to improve knowledge about opioid dependency and withdrawal and develop non-stigmatising care and communication practices. The e-learning module will be hosted on the Exchange Supplies training site with embedded attitude and knowledge evaluation measures assessed at pre- and post-completion. The module will draw on cultural safety principles to encourage practitioners to reflect on how power relations, social norms and inequity can impact health care opportunity and outcomes, including through the expression of their personal attitudes and beliefs.
d) A clinical guideline for managing opioid withdrawal, including continuity of care between community and hospital prescribers, and where necessary initiation or retitration of OST. The guideline will be developed through consultations with stakeholders, including people who use drugs, hospital staff, and representatives of national organisations, and will seek to reduce procedural barriers identified through a review of existing hospital policies ^24^.
e) An iHOST ‘champion’ role to support implementation of iHOST. The champion will support colleagues to adopt iHOST principles. iHOST champions will receive a role description, dedicated training in addition to the online module described above, and an ‘iHOST champion’ badge.

### Systematic review

We will do a systematic review of published studies that evaluate projects aiming to improve OST in acute hospital settings. This will include a structured search for studies using any design to evaluate relevant interventions and a narrative review of findings. The protocol is published on PROSPERO ^25^.

### Process evaluation

We will conduct a process evaluation at each site to examine intervention fidelity, acceptability, and perceived impact on practice. We will primarily use qualitative methods to explore implementation processes, intervention mechanisms and how these vary between sites.

The methods comprise:

1. Focus groups. These will be conducted before iHOST implementation with people who use opioids and hospital staff. The aim will be to develop a baseline understanding of each local context, including drug market dynamics, service accessibility for people who use drugs, hospital opioid withdrawal management practice and perceived quality of relationships between drug treatment and hospital services. One group will be held at each local community drug treatment service and intervention hospital (ie. a total of six groups).
2. In-depth interviews. These will be conducted at least two months after iHOST is launched, with hospital staff and inpatients who use opioids. The aim will be to assess acceptability and fidelity of implementation. Interviews will be informed by a topic guide including perceptions of the hospital culture, experiences of the intervention, care for patients who use opioids, communications between hospital and community services, and perceptions/experiences of opioid toxicity risk.
3. Observations. These will be conducted in each hospital site both prior to and during iHOST implementation. We will shadow clinical staff to observe the dynamics of care for people who use opioids in practice, and conduct observations during interview, training and focus group visits. Observations will be recorded as ethnographic field notes, with a focus on conveying detailed information about the context and culture of each hospital site and how this interacts with the iHOST implementation process.
4. Measuring intervention reach. We will get the number of advocacy cards distributed from local drug treatment services, numbers accessing the helpline, and numbers accessing the online module. The champions will be asked to keep a log of activities they carry out in support of intervention delivery (with support from the local iHOST lead).

***Analysis*.** We will transcribe all audio-recorded interviews and focus groups. Transcripts, interview field notes, and ethnographic observations will be uploaded into NVivo 12 for qualitative data management and analysis. We will conduct a thematic analysis, informed by constructivist grounded theory ^26^ principles. Grounded theory is an analytic method for studying processes, particularly useful for generating theory that can be applied in other sites and conditions. Coding will be implemented in a stepwise process, comprising open inductive coding, focused coding, category mapping, and theme development.

Qualitative data generated will take a number of forms (interviews, focus groups, ethnographic field notes), capture multiple perspectives (people who use drugs in and out of hospital, drug treatment and hospital care providers) and reflect different contexts (the three sites). We will ‘triangulate’ (compare/contrast) ^27^ these data to explore how and in what way the iHOST intervention works, for whom and in what context.

Measures of intervention reach will be described and assessed alongside the qualitative data to inform understandings of iHOST implementation in context, including barriers and facilitators to uptake of different intervention components, how this is reflected in patient and provider perceptions of the intervention and outcome measures for each site.

### Difference-in-difference study

***Study design.*** We will do a controlled study that will use data from iHOST hospitals and control hospitals (where iHOST is not implemented) to estimate the effect of iHOST on two outcomes: discharge against medical advice and emergency readmission within 28 days.

The study will use a difference-in-difference method ^28, 29^. This is a quasi-experimental design that aims to measure the effect of a change in clinical practice when the change in practice was not randomised. The method works by measuring patient outcomes in two groups (iHOST hospitals and control hospitals) and at two points in time. At the first point in time iHOST is not implemented in either group. At the second point in time, iHOST is only implemented in one group. The design is based on comparing the change in outcomes.

The purpose of including control hospitals is to account for background trends in outcomes that are common across hospitals. Examples include changes in the population (such as ageing), national policies, national restrictions related to COVID-19, and changes in drug supply. The design is more robust than an uncontrolled pre-post study, but less robust than a randomised experiment.

**Figure 2.**
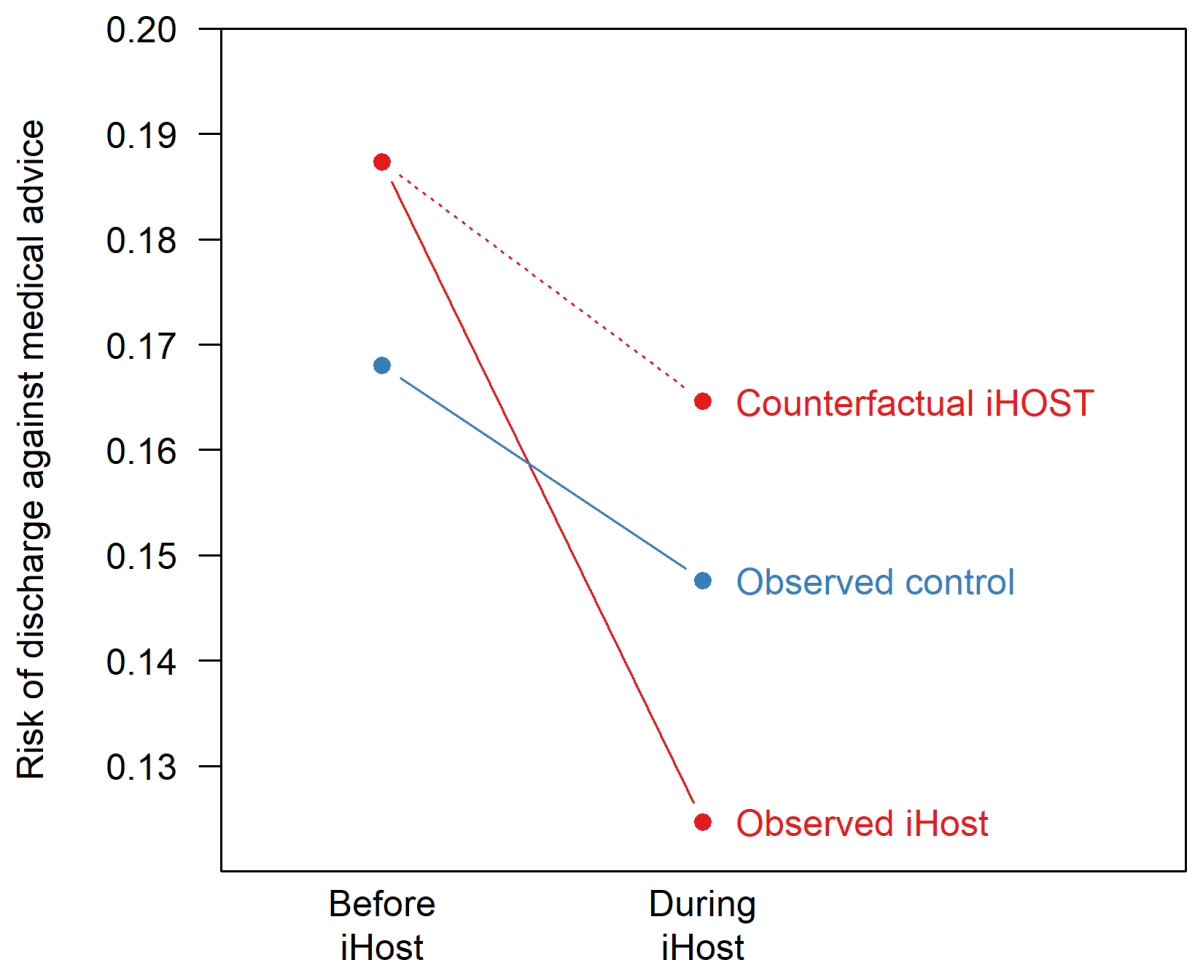
Illustrative difference-in-difference analysis.

A central assumption of the difference-in-difference method is that there are common trends in the outcome across participants. In the absence of the intervention, we assume that the change in outcome observed in the control group would have happened in the intervention group. In Figure 2, the ‘counterfactual iHOST’ risk is estimated using the trend in the control group, and the estimated effect of iHOST is the difference between the observed and counterfactual iHOST risks.

***Population.*** The study will use the Hospital Episode Statistics database, which includes all inpatient, outpatient, and A&E episodes at NHS hospitals in England ^30^.

The target population is hospital admissions for patients who may benefit from OST. Hospital Episode Statistics does not include records of OST (either in the community or in hospitals), and therefore the study will use a proxy of patients with records of opioid dependence. This will be defined as emergency hospital admissions (i.e., where the field ADMIMETH is 21-25, 2A, 2B, 2C, 2D, or 28) at acute hospitals in England, where the patient was (a) aged 18–64 years at admission; (b) the date of admission was between 12 months before and 12 months after the iHOST site’s implementation dates; and (c) the ICD-10 code F11 (‘mental and behavioural disorders due to use of opioids’) was recorded either at that admission or at another hospital episode for that patient in the preceding 12 months.

We will include patients from the three iHOST sites and from the 50 largest acute hospitals in England where iHOST is not being implemented (when size is defined by the number of patients meeting the criteria listed above). The reason for limiting the control group to the 50 largest hospitals is to exclude small hospitals that may have volatile trends in patient characteristics or outcomes.

***Analysis.*** iHOST will be implemented at different times at each of the three participating hospitals. This means that background trends in outcomes could be different, and the analysis will therefore be done separately for each hospital.

For analysis of discharge against medical advice, study entry will be at hospital admission. For analysis of readmission, study entry will be at discharge, such that patients who died during the index admission are excluded. We will use a mixed (hierarchical) linear model in which the dependent variable is a binary indicator of the outcome, and independent variables are an interaction term between two binary outcomes: time (i.e., before/after iHOST implementation) and iHOST site or control; a random intercept for the hospital site; and patient-level confounders/covariates. This approach to modelling is known as a ‘linear probability model’, and we have selected it to allow estimation of absolute difference in the risk of outcomes. Confounders will be age, sex, season, primary cause of admission, number of comorbidities, proportion of patients at the hospital who have COVID-19. We will fit the model using a restricted maximum likelihood method implemented in the R package lme4 ^31^. The effect of iHOST will be interpreted from the interaction between time and the control/intervention indicator. Definitions of study variables are included in Extended Data ^32^.

***Baseline data analysis.*** We extracted data from Hospital Episode Statistics for the calendar year 2021 to estimate the number of eligible patients and understand their characteristics. The results are summarised in Table 1. Hospitals are anonymised.

**Table 1.** Number and characteristics of patients with recent diagnoses of opioid dependence in the calendar year 2021, based on preliminary analysis of Hospital Episode Statistics.

Site	Number of patients	Discharge against medical advice N (%)	28-day readmission N (%)	Age (IQR)	Male N (%)	5+ comorbidities N (%)	% patients at hospital with COVID-19
iHOST sites							
A	349	70 (20.1)	101 (28.9)	46 (36-54)	244 (69.9)	143 (41.0)	10.1
B	662	149 (22.5)	214 (32.3)	43 (38-47)	489 (73.9)	195 (29.5)	6.9
C	624	116 (18.6)	148 (23.7)	43 (37-50)	392 (62.8)	182 (29.2)	4.8
Control hospitals							
All	24604	4048 (16.5)	7001 (28.5)	43 (37-50)	16425 (66.8)	6718 (27.3)	7.9
1	893	61 (6.8)	322 (36.1)	42 (35-49)	535 (59.9)	97 (10.9)	6.1
2	818	102 (12.5)	156 (19.1)	40 (35-47)	584 (71.4)	138 (16.9)	8.3
3	728	103 (14.1)	183 (25.1)	45 (37-52)	458 (62.9)	134 (18.4)	9.0
4	732	191 (26.1)	265 (36.2)	45 (38-50)	533 (72.8)	208 (28.4)	6.7
5	739	92 (12.4)	249 (33.7)	43 (37-50)	445 (60.2)	185 (25.0)	5.8
Etc …							

**Figure 3.**
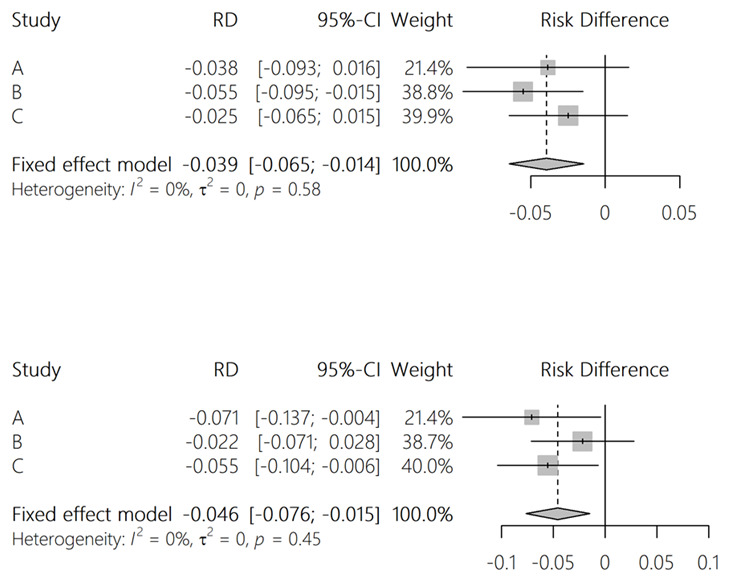
Example results from a single simulation of the difference-in-difference study.

***Power.*** To estimate power, we simulated the study assuming that iHOST reduces the risk of each outcome by 5 percentage points (for example from 20% to 15%). For the “pre iHOST” data, we used the calendar year 2021 (summarised in Table 2). For the "post iHOST” data, we used same dataset; but with outcomes randomly generated where the risk of each outcome was the hospital-specific risk from 2021, reduced by 5 percentage points for the iHOST sites. An example of a single simulation is shown in Figure 3. We repeated this simulation 1,000 times and calculated the proportion of simulations in which a significant result was found (ie. p <0.05). This suggested good power to detect a risk reduction of 5 percentage points when results from the three hospitals are pooled, but low power at individual sites. Code for the power analysis is available at https://github.com/danlewer/ihost/tree/main/power.

**Table 2.** Power of the iHOST study to detect an absolute reduction in outcomes of five percentage points.

	Discharge against medical advice	28-day emergency readmission
Site A	0.475	0.228
Site B	0.839	0.442
Site C	0.837	0.376
Pooled effect	>0.999	0.921

### OST process measures

Participating hospitals will use local clinical data to report measures of the quality of OST provision (‘process measures’). We have worked with information specialists and clinicians at University College London Hospital NHS Foundation Trust to develop the following measures:

1. The number of patients administered methadone. Most patients provided with OST in acute hospitals in the UK are given methadone; and additionally, methadone is rarely given for other indications. Therefore, it is a useful indicator of the quantity of OST provided; while avoiding the need to consider the indication and formulation.
2. Duration between decision to admit and administration of methadone, reported as (a) the proportion that is 24 hours or greater; and (b) the median and interquartile range.
3. The proportion of patients administered at least 20mg of methadone in one 24-hour period; reported as a proportion of patients who are administered methadone and admitted for at least 24 hours.
4. The proportion of potentially eligible patients who are provided with OST, defined as the proportion of inpatients with a discharge diagnosis of ICD-10 F11 (‘opioid related disorders’) who were administered methadone or buprenorphine during their admission.
5. The proportion of patients who are potentially eligible for OST that discharge against medical advice, defined as the proportion of inpatients with discharge diagnosis of ICD-10 F11 (‘opioid related disorders’) who discharge against medical advice.
6. Possible OST-associated overdoses, defined as inpatients who are administered naloxone after an administration of methadone or buprenorphine.

Depending on the feasibility of extracting these data, each iHOST site will calculate each measure for quarterly periods (i.e., every three months) for time periods before and during iHOST implementation. The sites will nominate a group of staff (for example medical staff, pharmacists, and information specialists) to review the indicators every three months. We will also do an uncontrolled before/after analysis to test if the indicators change after implementation of iHOST.

### Health economic study

We will perform a cost-consequence analysis of the iHOST intervention compared to not using it, in terms of cost per OST prescription, cost per discharge against medical advice event prevented and cost per 28-day emergency readmission prevented. Analysis will use an NHS cost perspective and include each of the main elements of the intervention (MyMedsCard, helpline, OST champion and the e-learning module) as well as the costs of OST prescription within the hospital setting. As the opioid withdrawal management policy guideline will not need to be developed again on roll-out of the intervention, we assume this cost to be negligible and will therefore not conduct a detailed cost estimate of the policy development. We will include the cost savings for 28-day emergency visits prevented, but will exclude the costs of unrelated hospital visits.

### Safety

iHOST may involve changes to OST prescribing in participating hospitals. Changes to guidelines and clinical processes will be agreed through existing clinical governance structures at participating hospitals and will become part of usual care. Potential safety issues will be identified through these processes. Where events occur that may be related to the safety of new OST pathways, the events will be reviewed by the participating hospital.

### Ethics and approvals

Ethical approvals have been received from the NHS Health Research Authority on 8 September 2022 [Ref 133022], the Camden and Islington NHS Research Ethics Committee on 31 May 2022 [Ref 22/LO/0370] and the LSHTM Observational Research Ethics Committee on 18 July 2022 [Ref 27895]. Local research and development approvals have been obtained from each hospital site and participating drug treatment service.

The difference-in-difference study was approved by the UK Health Security Agency Research Ethics and Governance Group on 29 March 2022 [R&D ref 497].

## Discussion

Poor OST in acute hospitals is a barrier to effective care for patients who use illicit opioids. This is clear from our research in the UK ^33– 35^, engagement with affected groups, and international evidence ^8, 12, 13, 22, 36, 37^. Poor OST can cause physical and psychological distress and result in treatment interruption or patients leaving hospital to obtain illicit drugs ^8, 12^. People experiencing opioid withdrawal can be perceived as challenging by healthcare workers. Improving care for patients who use illicit opioids and their feeling of safety could be beneficial for both patients and hospital staff. Although there are well-documented problems with OST in hospitals ^6– 10^, this has not led to evaluated interventions in the UK.

Barriers to better OST have been investigated, and qualitative evidence has found procedural and attitudinal barriers. For example, medical professionals often have negative attitudes towards patients who use opioids, which may impact care ^10, 38^. Fear and experience of drug withdrawal are key factors for discharge against medical advice ^12, 39^, which is associated with hospital readmission and increased mortality risk ^40– 44^.

iHOST is designed to address these barriers using a multi-component intervention that addresses procedures, knowledge, and attitudes. It is an applied research project in which the intervention is developed in a real-world setting. If successful, it should improve management of withdrawal and reduce delays in the prescription of OST. We anticipate that improvement of OST will lead to improved patient care, improved patient experiences, a reduction in discharge against medical advice, and reduced re-admissions.

We will evaluate the intervention using a pragmatic mixed-methods approach. This will provide a holistic understanding of the intervention’s costs and benefits. We chose this approach over a cluster randomised trial for three reasons. First, such a trial would be expensive. Second, the barriers to implementation are different in each hospital and iHOST is focused on developing an intervention that is suitable for each site, rather than a consistent intervention. Third, we do not necessarily expect a clear effect on a single primary outcome. Given the marginalisation of the patient group and the long histories of adversity that many have experienced, it is possible that many will still leave hospital against medical advice despite better OST and better patient care. Our engagement with patients and staff suggests that better OST could reduce suffering and increase trust between staff and patients even if primary outcomes in our quantitative study do not appear to change significantly. We wanted to capture these holistic outcomes.

Key limitations are:

1. **Process evaluation.** (a) The research team involved in intervention development will also oversee the process evaluation. This might inhibit some staff and stakeholders from expressing robust critique of the intervention, particularly if interviewed by researchers recognisable from e-learning training videos. A different team member will, therefore, conduct most of the qualitative interviews after the intervention has launched at the evaluation sites. (b) We will recruit inpatients who use opioids through hospital staff. This is crucial to ensure patients are well enough to consent, are present on the ward, and meet inclusion criteria (ie, use opioids). This can however, facilitate ‘gatekeeping’, whereby patients likely to provide a favorable impression of the hospital or intervention are selected for interview. In building relationships with hospital staff, we will stress the need to talk with a diversity of patients and the learning to be gained from understanding less positive experiences. (c) Observations include active shadowing of hospital staff on ward rounds. The presence of the researcher, and their association with the intervention, can impact staff-patient dynamics observed. Detailed field notes will be taken for all site visits, including interviews and staff training sessions, to provide additional context.
2. **Controlled difference-in-difference study.** (a) No data will be available on OST prescriptions, either in the community or in hospital. Ideally, our study would include patients who had a community OST prescription prior to admission. Instead, we will use patients with a diagnosis of ‘opioid dependence’ as a group that may benefit from iHOST. This may dilute the effect of iHOST because some participants will not have prescriptions of OST; (b) the study will not capture any effect on patients’ propensity to seek treatment. Part of the rationale for iHOST is that people who are dependent on opioids avoid or delay hospital treatment because they anticipate poor OST. This study investigates outcomes after admission and will not capture any effect on patients’ propensity to seek treatment in hospital. If iHOST leads to more patients seeking hospital care or presenting earlier, this may cause residual confounding because patients after implementation will differ from those before; (c) The study will only estimate effects on discharge against medical advice and readmission. These outcomes are based on previous research, patient and public involvement, and the availability of data in HES. Other important outcomes may be affected by iHOST, such as the quality of medical treatment, patient satisfaction, continuation of OST in the community, and use of illicit drugs. Other parts of the evaluation seek to understand broader outcomes. A more detailed list of limitations for the difference-in-difference study is provided in Extended Data ^32^.
3. **OST process measures**. These measures are intended to support iHOST sites to learn about the quality of OST in their setting and identify opportunities for ongoing improvement. They are not a robust approach to estimating the impact of the intervention, as a change in these measures could be attributed to many factors other than the intervention, such as changing patient demographics, changing use of OST (eg. if iHOST leads to different types of patients being given OST), and wider healthcare policies.
4. **Health economic study.** We may not be able to accurately estimate the difference in methadone prescriptions. In many cases methadone may have been prescribed without the iHOST protocol, but having the protocol in place may mean that it is prescribed more quickly. We will do sensitivity analysis around this, assuming ranges in counterfactual prescription rates without the iHOST protocol in place. Costs included in the economic evaluation will be specific to sites where the study is operating and may not be generalisable to other settings or implementation of iHOST at large scale. We are unable to do any modelling of long-term outcomes, and therefore cost and effect estimates may not include important future events.

## Conclusions

This work aims to recognise that patients who use illicit opioids are often vulnerable and require support in their journey through the healthcare system. iHOST aims to promote a culture change in which patients who use opioids feel confident to access medical care. This can also benefit providers and staff who sometimes feel unconfident working with this group and may consider them ‘challenging’. iHOST training will provide skills in de-escalation strategies and culturally safe communication. The work with clinical policies will address procedural barriers to OST provision. We hope these changes will improve medical care and reduce health inequality.

## Data Availability

No data are associated with this article. LSHTM Research Online: Extended data for ‘Improving hospital-based opioid substitution therapy (iHOST): protocol for a mixed-methods evaluation’, https://doi.org/10.17037/PUBS.04671913 ^32^ This project contains the following extended data: Definitions of variables for difference-in-difference study Limitations for difference-in-difference study Data are available under the terms of the Creative Commons Attribution 4.0 International license (CC-BY 4.0)
