# Supplementary material for: Improving hospital-based opioid substitution therapy (iHOST): protocol for a mixed-methods evaluation
Source: NIHR Open Res. 2024 Nov 6;4:10. Originally published 2024 Mar 13. [Version 2] doi: 10.3310/nihropenres.13534.2 (PMC11576563; doi:10.3310/nihropenres.13534.2)
Supplement: Peer review history

# Reviewer report: Reviewer response for version 2

**DOI:** 10.3310/nihropenres.14984.r33680

**Published:** 2024-11-21

**Authors:** Theresa Caruana

**Affiliations:** 1 UNSW Sydney, Sydney, Australia

This is a well-designed protocol for evaluating the implementation of an intervention that aims to improve the outcomes and experiences of hospital admissions by people who receive opioid substitution therapy. The intervention has been co-designed with people who use opioids and hospital staff and is intended to increase knowledge and support in responding to this patient group appropriately. The scope of the described evaluation strategies is impressive. The analytical techniques proposed are very suitable and are described in good detail, particularly with respect to their limitations. There appear to be a number of systemic difficulties that have affected the evaluation design, including the lack of ability to calculate OST prescriptions in the community and the proportions of unmet treatment needs in hospital admissions. The study does well to consider and work around these in detailing its proposed methods.  

Is the study design appropriate for the research question?

Yes

Is the rationale for, and objectives of, the study clearly described?

Yes

Are sufficient details of the methods provided to allow replication by others?

Yes

Are the datasets clearly presented in a useable and accessible format?

Not applicable

Reviewer Expertise:

Opioid agonist treatment in NSW (Australia) pharmacies; health systems research; qualitative study design; quantitative study design

I confirm that I have read this submission and believe that I have an appropriate level of expertise to confirm that it is of an acceptable scientific standard.

# Reviewer report: Reviewer response for version 2

**DOI:** 10.3310/nihropenres.14984.r33375

**Published:** 2024-11-19

**Authors:** Sandra A Springer

**Affiliations:** 1 Yale School of Medicine, Connecticut, USA

Yes I reviewed and approve the revised version

Is the study design appropriate for the research question?

Partly

Is the rationale for, and objectives of, the study clearly described?

Yes

Are sufficient details of the methods provided to allow replication by others?

Partly

Are the datasets clearly presented in a useable and accessible format?

Not applicable

Reviewer Expertise:

Opioid use disorder treatment in hospital, community and carceral systems including buprenorphine both sublingual and injectable forms as well as extended-release naltrexone; access to services via linkage care models including patient navigators and mobile health unit provision of care for persons who use drugs ; HIV prevention and treatment; Infectious disease integrated with SUD care; mobile pharmacies.

I confirm that I have read this submission and believe that I have an appropriate level of expertise to confirm that it is of an acceptable scientific standard.

# Reviewer report: Reviewer response for version 1

**DOI:** 10.3310/nihropenres.14692.r33051

**Published:** 2024-10-08

**Authors:** Chris Tremonti

**Affiliations:** 1 Alcohol and Drug Services St Vincent’s Hospital, University of Sydney, Sydney, Australia

Thank you to the authors for the opportunity to review this article, which is in fact indexing of a study protocol. As a consequence, it is somewhat unusual to review as the authors will no doubt have undergone extensive review of their protocol during the process of seeking ethics. I am also unqualified to review some of the more novel statistical methodologies employed for the study. While this does appear scientifically robust, I would suggest if the journal had concerns to seek review from a biostatician, but I suspect one has been involved in the write-up, and likely review the protocol during the ethics process.

 I do not think it is worth a significant reevaluation of the protocol here. Nonetheless I will mention some minor limitations that may be worth commenting upon, and could be added under the limitations already listed:

1. 'Patients who may benefit from OST' – a limitation of using the ICD-11 diagnostic criteria of opioid use disorder is that that those not forthcoming in their opioid use may not be captured. In hospitals without ihost – where stigma may be higher against those who use opioids – there may therefore be less people captured by this criteria, and a ‘falsely high’ proportion of people receiving appropriate treatment. I’m unsure if I have a solution to that problem, it may just be worth discussing.
2. I am unclear why the authors are only capturing those administer methadone. I do appreciate that buprenorphine is now commonly being used for pain management, but not typically at the doses seen in OST. Furthermore in my jurisdiction it is commonly used in the form of suboxone or subutex, while temgesic is used for pain. I do wonder if patients on buprenorphine could be captured, perhaps with a criteria of it needing to be in a specific form, or on a daily dose >8mg (which would be unusual in pain management).
3. The limitation of treatment bias warrants a small discussion too - those hospitals with iHOST may be geared towards the success of its implementation.

In spite of these pedantic points, this is a very worthy and ambitious study being undertaken, and I wish the authors the best with it.

 I would add to the journal that given my lack of expertise with both lived experience and biostatistics, it may be worth review from those with expertise in these areas

Is the study design appropriate for the research question?

Yes

Is the rationale for, and objectives of, the study clearly described?

Yes

Are sufficient details of the methods provided to allow replication by others?

Yes

Are the datasets clearly presented in a useable and accessible format?

Yes

Reviewer Expertise:

Addiction medicine

I confirm that I have read this submission and believe that I have an appropriate level of expertise to confirm that it is of an acceptable scientific standard.

# Response

**Published:** 2024-11-04

**Authors:** Dan Lewer

11. 'Patients who may benefit from OST' – a limitation of using the ICD-11 diagnostic criteria of opioid use disorder is that that those not forthcoming in their opioid use may not be captured. In hospitals without ihost – where stigma may be higher against those who use opioids – there may therefore be less people captured by this criteria, and a ‘falsely high’ proportion of people receiving appropriate treatment. I’m unsure if I have a solution to that problem, it may just be worth discussing.

*Response: We agree, this is a potential source of bias. We have added the following text to the list of limitations in our supplementary file: “* ***Use of ICD-10 codes may differ between iHOST and control sites*** *. We will use ICD-10 diagnoses (F11, Mental and behavioural disorders due to use of opioids) to identify eligible patients. The use of these codes may differ between hospitals. Bias may occur if iHOST sites become more likely to identify opioid dependence after implementation of the intervention. For example, this scenario may lead to a lower average severity of opioid dependence because the hospital may have a lower threshold for identifying opioid dependence, and therefore an inflated effect estimate. This is just an example of a potential bias resulting from changing use of ICD-10 codes.”*

 12. I am unclear why the authors are only capturing those administer methadone. I do appreciate that buprenorphine is now commonly being used for pain management, but not typically at the doses seen in OST. Furthermore in my jurisdiction it is commonly used in the form of suboxone or subutex, while temgesic is used for pain. I do wonder if patients on buprenorphine could be captured, perhaps with a criteria of it needing to be in a specific form, or on a daily dose >8mg (which would be unusual in pain management).

*Response: Please also see comments #7 and #8. In our context, buprenorphine is rarely used for OST, and in addition detailed prescribing data is typically difficult to capture in a structured format. In our partner hospitals it would likely require reading free-text notes for each patient prescribed buprenorphine. ie. The information specialists at the hospitals can query a database to identify patients prescribed buprenorphine, but they cannot determine what product and dose was used. The majority who are prescribed buprenorphine probably would not be receiving OST.*

 13. The limitation of treatment bias warrants a small discussion too - those hospitals with iHOST may be geared towards the success of its implementation.

*Response: We agree and have added the following text under Limitations/process evaluation: “(d) The three hospitals participating in iHOST may be more motivated or otherwise optimised for successful implementation. Recommendations for roll-out will need to account for potential additional barriers such as the absence of a motivated senior ‘champion’.”*

# Reviewer report: Reviewer response for version 1

**DOI:** 10.3310/nihropenres.14692.r31990

**Published:** 2024-06-18

**Authors:** Sandra A Springer

**Affiliations:** 1 Yale School of Medicine, Connecticut, USA

This is a protocol paper for a mixed methods evaluation to improve “Opioid substitution treatment (OST)”  in 3 acute hospitals in England.

 Importantly this study was informed by discussions with key collaborators that included persons who use opioids as well as hospital staff.

 This study aims to establish an identification card for patients to give to hospital clinicians to confirm their OST, a helpline for patients and staff, an online training module for staff, a clinical guideline for managing opioid withdrawal in the hospital and will develop roles for a champion at each hospital. Primary outcome is “discharge against medical advice and emergency readmission within 28days .‘

 The investigators plan to do a comparison of changes in outcomes for patients at iHOST hospital sites with changes in outcomes from patients at control hospital sites.

 The importance of the subject matter and study aims are well delineated. Many persons admitted to hospitals with OUD are not identified and can go without treatment for OUD and lead to withdrawal from opioids and early discharges as well as increases in death from overdose and readmissions as well related to poorly treated/untreated other medical problems such as serious injection related infections.

 Here the authors use the term ‘opioid substitution treatment’ (OST), however in the United States the terms are not used any more as it has perpetuated myths of treatment of OUD with opioid agonists as a substitution of one opioid for another. Thus, American Society of Addiction medicine (ASAM) and SAMHSA  and NIDA and other groups now refer to OUD medication treatment as medication treatment of OUD (MOUD) and sub-categorizes the forms of MOUD as opioid agonist-based treatments, methadone and buprenorphine, and opioid antagonist treatments, extended-release naltrexone. I understand this is a UK specific study, but is a concern I voice for all persons globally and is in particular for those in the U.S.

 Outcomes of interest are patient self- discharges and readmissions. The authors use the term ‘leaving against medical advice (AMA)’ and this term is also stigmatizing and not used in the literature any longer in the U.S., it is described as’ patient self-discharge’.

 Strength of this proposed study is that it was informed by persons with lived experience including those with OUD on MOUD or here opioid agonists ( or what authors report as OST) and  staff as well as with culturally specific safety principles .

 The overall aims are to improve OST use in hospital settings and reduce "delayed presentation". I found this term a bit difficult to understand- the authors mean delayed treatment with OST or delayed specifically continuation of OST as they are specifically only identifying persons with OUD on OST in community who are admitted to hospitals thus  Would suggest possibly editing this term or meaning of term under Aims and Objectives.

 The study is focusing on those who are on 'OST in the community' and helping them ensure their caregivers in the hospital are aware – hence the ID card. I wonder however if more could be done here including identifying persons with OUD who have not been started on OST or those who previously had been but were no longer retained on OST.  Hospitalizations are reachable moments where persons can be initiated or reinitiated on treatment for OUD .  In the U.S there is no universal diagnosis of SUD/OUD and many are admitted and OUD is not identified leading to missed opportunities to engage/ initiate MOUD and link to care in the community and thus with goal to reduce overdose and other associated harms like HIV, HCV , injection related infections, depression/ suicide etc. 

 Concerns about the ID card proposed in this study I have are the potential for staff/ patients misplacing it and leading to confidentiality breaches. Is it possible to make electronic for persons smart phones  or other way to assure decreasing chance of loss of confidentiality?

 ‘Difference in difference Study ‘section:

 Authors plan to do a ‘controlled study’design using data from iHOST hospitals and control hospital to estimate the effect of iHOST on AMA discharge and ER readmission within 28 days.

 Concerns for this design, is that there will be significant intra-group and inter-group differences that could affect the outcomes. For example, staff differences (bias, experience etc)  and hospital/ setting differences. Is it possible instead to compare the intervention to treatment as usual in the 3 hospitals?

 I am not familiar with this type of study design and thus would request a person with such design familiarity to review this section as well as the power analysis.  My concerns are: the many differences of the particular patients at each sites as well as staff and hospital settings/ experience etc with addiction and support services . The analysis of the data from this study will have to balanced as much as possible for such differences and acknowledge this as a limitation of the final analysis as of course not everything will be able to be accounted for.  Would suggest a statistician who has familiarity with this type of evaluation / study design/ analytic plan to comment on this concern.

 ‘Ost process measures’ section: .

 Not clear but seems like only methadone is to be evaluated ,  not buprenorphine.  Not explained why hospitals in UK use methadone more than buprenorphine. Would make this more clear in the intro/ abstract and methods sections as instead of what they call ‘OST” it is  only methadone.

 For methadone, would evaluate the mean dose of methadone. 40 mg is sufficient to treat some forms of opioid withdrawal but higher doses are needed for fentanyl withdrawal and of course for maintenance treatment and reduction in craving .Much higher doses over 90 and 100 mg are required to treat heroin and fentanyl addiction . We do know that persons who are not adequately dosed will be more likely to experience craving/ withdrawal and leave against medical advice. Thus the dose of methadone could affect the planned primary outcomes of ‘ patient leaving against medical advice’. If buprenorphine is also included as I would suggest, then also the mean dose should be evaluated and at least 16 mg is recommended to reduce craving etc and up to 32 mg ; also include long acting injectable buprenorphine monthly formulations now available that could be started in hospital settings or may have started in the community setting and continued in the hospital setting especially upon discharge ( sublocade 300mg and 100mg doses where 300 mg continuation has been found to improve retention among those who inject heroin/ fentanyl; and now Brixadi available at different doses and weekly/ monthly injections that are similar to the SL doses). 

 Other factors that could affect outcomes include comorbid other substance use like stimulants that can interfere with MOUD /OST use/access/ continuation/ retention as well as lead to withdrawal along with withdrawal from alcohol that if undiagnosed could lead to leaving against medical advice and increase hospital readmissions .. thus there needs to be a careful understanding of the population characteristics to see if persons enrolled in the trial have co-occurring other su/ sud that could affect their primary outcomes . Further, other factors that may lead to a difference in outcomes include other comorbid  psychiatric conditions, severity of illness upon hospital admission ( Elixhauser severity index could be added); pain ; housing status  etc that could impact ability to stay in hospital outside of whether on MOUD or not ( or here OST)- and specifically especially for pain - is there a difference in opioid agonist methadone versus partial agonist buprenorphine , dose etc and change in pain .. acute pain possibly related to hospital medical condition that may affect management of the treatment of OUD as well. 

 Safety

 Highly recommend that the investigators assess adverse events and serious adverse events as is standard in NIH reporting / etc .

Is the study design appropriate for the research question?

Partly

Is the rationale for, and objectives of, the study clearly described?

Yes

Are sufficient details of the methods provided to allow replication by others?

Partly

Are the datasets clearly presented in a useable and accessible format?

Not applicable

Reviewer Expertise:

Opioid use disorder treatment in hospital, community and carceral systems including buprenorphine both sublingual and injectable forms as well as extended-release naltrexone; access to services via linkage care models including patient navigators and mobile health unit provision of care for persons who use drugs ; HIV prevention and treatment; Infectious disease integrated with SUD care; mobile pharmacies.

I confirm that I have read this submission and believe that I have an appropriate level of expertise to confirm that it is of an acceptable scientific standard, however I have significant reservations, as outlined above.

# Response

**Published:** 2024-11-04

**Authors:** Dan Lewer

1. Here the authors use the term ‘opioid substitution treatment’ (OST), however in the United States the terms are not used any more as it has perpetuated myths of treatment of OUD with opioid agonists as a substitution of one opioid for another. Thus, American Society of Addiction medicine (ASAM) and SAMHSA  and NIDA and other groups now refer to OUD medication treatment as medication treatment of OUD (MOUD) and sub-categorizes the forms of MOUD as opioid agonist-based treatments, methadone and buprenorphine, and opioid antagonist treatments, extended-release naltrexone. I understand this is a UK specific study, but is a concern I voice for all persons globally and is in particular for those in the U.S.

*Response: We are aware of these developments in language and perceptions about the term “substitution”. We named our project “iHOST” (improving hospital opiate/opioid substitution therapy” together with patients and clinicians. This reflects historical terminology and vernacular in these groups, with “OST” remaining the best-recognised term in the UK. While we would be very willing to change the terminology in this article, it would make the text inconsistent with the project name. We also note that no term for these medicines is universally acceptable, for example some people consider the word “disorder” to be pejorative and therefore prefer not to use “medication treatment of opioid use disorder”. For consistency with the project name “iHOST”, we have retained the use of “OST” in this article, however in the abstract and at the start of the introduction we have added: “Opioid substitution therapy (also known as opioid agonist therapy and medication treatment of opioid use disorder)”.*

1. Outcomes of interest are patient self- discharges and readmissions. The authors use the term ‘leaving against medical advice (AMA)’ and this term is also stigmatizing and not used in the literature any longer in the U.S., it is described as’ patient self-discharge’.

*Response: We agree and have changed our terminology to “patient-direct discharge”.*

1. The overall aims are to improve OST use in hospital settings and reduce "delayed presentation". I found this term a bit difficult to understand- the authors mean delayed treatment with OST or delayed specifically continuation of OST as they are specifically only identifying persons with OUD on OST in community who are admitted to hospitals thus  Would suggest possibly editing this term or meaning of term under Aims and Objectives.

*Response: Our sentence as originally written was confusing, and we have changed it to “iHOST aims to improve OST in hospital settings to: (1) reduce barriers to hospital presentation and reduce delayed presentations, (2) improve care, (3) reduce patient-directed discharges, and (4) reduce emergency readmissions.” The first aim relates to patients who should go to A&E/ED but do not because they believe they will not be treated well. Our qualitative and patient engagement work suggests this happens often. A typical scenario would be a patient with a skin and soft tissue infection waiting until they have symptoms of systemic infection before going to hospital. We are only able to evaluate aims 3 and 4 quantitatively, and other aspects of the projects consider the other aims. Hopefully this is clearer, but please let us know if you have further feedback.*

1. The study is focusing on those who are on 'OST in the community' and helping them ensure their caregivers in the hospital are aware – hence the ID card. I wonder however if more could be done here including identifying persons with OUD who have not been started on OST or those who previously had been but were no longer retained on OST.  Hospitalizations are reachable moments where persons can be initiated or reinitiated on treatment for OUD .  In the U.S there is no universal diagnosis of SUD/OUD and many are admitted and OUD is not identified leading to missed opportunities to engage/ initiate MOUD and link to care in the community and thus with goal to reduce overdose and other associated harms like HIV, HCV , injection related infections, depression/ suicide etc. 

*Response: This is a fair observation, and iHOST does largely focus on continuity of OST. The UK context is different to North America since between half and three-quarters of patients who are dependent on opioids already have OST prescriptions and hospital staff can have difficulty determining the daily dose and when the next dose is due. Therefore, medicine reconciliation and continuity of doses is a major consideration in the UK. iHOST does also aim to improve initiation of OST, and the text under the section “The Intervention” says that iHOST “primarily aims to improve continuation of OST from community to hospital settings but will also inform OST initiation and discharge planning for patients who were not already in receipt of community-based OST.” The specific approaches are not detailed in the protocol, however the policies/protocols and training materials at participating hospitals address initiation of OST and these will be published in due course.*

1. Concerns about the ID card proposed in this study I have are the potential for staff/ patients misplacing it and leading to confidentiality breaches. Is it possible to make electronic for persons smart phones  or other way to assure decreasing chance of loss of confidentiality?

*Response: This issue was also raised when we developed the card. The card does not include the patient’s details, and only includes information about the importance of timely OST, and contact details for the patient’s prescriber. You can see images of the card here:* *https://www.lshtm.ac.uk/research/centres-projects-groups/ihost#about* *. We did explore electronic versions but could not design a feasible solution – problems included the fact that many patients do not have the same phone / online accounts over time, and technical integration with prescribers’ clinicals systems was challenging.*

1. (Concerning the difference-in-difference analysis) Concerns for this design, is that there will be significant intra-group and inter-group differences that could affect the outcomes. For example, staff differences (bias, experience etc)  and hospital/ setting differences. Is it possible instead to compare the intervention to treatment as usual in the 3 hospitals? […] My concerns are: the many differences of the particular patients at each sites as well as staff and hospital settings/ experience etc with addiction and support services . The analysis of the data from this study will have to balanced as much as possible for such differences and acknowledge this as a limitation of the final analysis as of course not everything will be able to be accounted for.  Would suggest a statistician who has familiarity with this type of evaluation / study design/ analytic plan to comment on this concern.

*Response: The design is observational and there is always the possibility of residual confounding. In the difference-in-difference design, the control group (ie. hospitals that did not implement iHOST) are intended to control background time trends in the outcome that are common across all hospitals. Differences in the baseline outcome rate (eg. if hospitals in the control group have lower overall rates of patient-directed discharge) would not bias the study. A bias would occur if the trend in outcomes over time was different; for example if patient-directed discharge increased in the control hospitals, but would not have increased in the iHOST hospitals in the counterfactual scenario that we did not implement iHOST. In this scenario, the estimated effect of iHOST would be overestimated. The assumption that the time trends are the same in the iHOST and control groups is known as the “parallel trends assumption”. The robustness of this assumption cannot be directly observed, since that would require observation of the counterfactual scenario in which iHOST hospitals did not implement iHOST. However, this design does mean that more general differences in patient profiles, availability of addiction and support services, etc., should not cause major bias. This is in fact the case, and our baseline data suggest that patient-directed discharge is more common in iHOST hospitals than in control hospitals (see our presentation of the design to the NIHR Statistics Conference -* *https://www.youtube.com/watch?v=rC9GJya6h6Y&list=LL&index=9* *). If these factors change differentially between the iHOST and control groups, that could cause bias. Changes over time in measured factors are controlled. For example, if the patient age, primary reason for admission, or comorbidities change in one group, that would be controlled. Similarly, if some hospitals are more affected by COVID-19, that would also be controlled. The design was developed by methodologists on the study team and reviewed by an independent statistician (Professor Ben Armstrong at LSHTM) as part of our study approvals.*

1. ‘Ost process measures’ section: Not clear but seems like only methadone is to be evaluated,  not buprenorphine.  Not explained why hospitals in UK use methadone more than buprenorphine. Would make this more clear in the intro/ abstract and methods sections as instead of what they call ‘OST” it is  only methadone.

*Response: buprenorphine is used as OST in the UK, but much less frequently than in North America. The reasons for this include: (1) the cohort in treatment for opioid dependence in the UK is older and many started treatment before buprenorphine was available; (2) there are fewer regulatory barriers to using methadone in the UK. We do not have data on the proportion of individuals who have methadone vs. buprenorphine prescriptions, but anecdotally very little buprenorphine is currently prescribed for OST in our participating hospitals. In many UK hospital policies on management of substance dependence, buprenorphine is not even mentioned (* *https://pmc.ncbi.nlm.nih.gov/articles/PMC9007696/* *). We have added the following sentence to the introduction: “*While data on the relative use of methadone and buprenorphine in OST in the UK is not readily available, some hospital guidelines for management of substance dependence do not mention buprenorphine.”

1. For methadone, would evaluate the mean dose of methadone. 40 mg is sufficient to treat some forms of opioid withdrawal but higher doses are needed for fentanyl withdrawal and of course for maintenance treatment and reduction in craving .Much higher doses over 90 and 100 mg are required to treat heroin and fentanyl addiction . We do know that persons who are not adequately dosed will be more likely to experience craving/ withdrawal and leave against medical advice. Thus the dose of methadone could affect the planned primary outcomes of ‘ patient leaving against medical advice’. If buprenorphine is also included as I would suggest, then also the mean dose should be evaluated and at least 16 mg is recommended to reduce craving etc and up to 32 mg ; also include long acting injectable buprenorphine monthly formulations now available that could be started in hospital settings or may have started in the community setting and continued in the hospital setting especially upon discharge ( sublocade 300mg and 100mg doses where 300 mg continuation has been found to improve retention among those who inject heroin/ fentanyl; and now Brixadi available at different doses and weekly/ monthly injections that are similar to the SL doses). 

*Response: This is a good idea and we will do it if possible, however in practice we are not able to estimate the mean dose at our pilot site (University College Hospital, London). Reporting the dose per patient seems straightforward, however there are various factors that make it difficult: (1) patients often receive multiple doses per day, so the dose must be calculated over an arbitrary period, eg. 24-hour intervals after their admission time; (2) the admission time can have a number of definitions, and the dose is sensitive to the choice; (3) the prescribed and administered schedule may differ, for example some doses may be late; (4) the prescribed schedule may only be available in free-text notes; (5) some patients have duplicate dose data that is difficult to validate. Although similar challenges are likely to arise at our two other sites, we will report summary measures of the dose if possible, and have edited the text to say: “3. The dose, defined as (a) the proportion of patients administered at least 20mg of methadone in one 24-hour period; reported as a proportion of patients who are administered methadone and admitted for at least 24 hours, and (b) the mean and distribution of administer doses across 24-hour following admission.”*

1. Other factors that could affect outcomes include comorbid other substance use like stimulants that can interfere with MOUD /OST use/access/ continuation/ retention as well as lead to withdrawal along with withdrawal from alcohol that if undiagnosed could lead to leaving against medical advice and increase hospital readmissions .. thus there needs to be a careful understanding of the population characteristics to see if persons enrolled in the trial have co-occurring other su/ sud that could affect their primary outcomes . Further, other factors that may lead to a difference in outcomes include other comorbid  psychiatric conditions, severity of illness upon hospital admission ( Elixhauser severity index could be added); pain ; housing status  etc that could impact ability to stay in hospital outside of whether on MOUD or not ( or here OST)- and specifically especially for pain - is there a difference in opioid agonist methadone versus partial agonist buprenorphine , dose etc and change in pain .. acute pain possibly related to hospital medical condition that may affect management of the treatment of OUD as well. 

*Response: We agree – these are all important factors that could affect patient-directed discharge and readmission. From a quantitative perspective, these are potential confounders and may affect the study if time-trends in these factors differ between iHOST and control sites (please see comment #6). From a qualitative perspective, we will be gathering evidence about factors that affect access to OST and patient outcomes via interviews with staff and clinicians.*

1. Highly recommend that the investigators assess adverse events and serious adverse events as is standard in NIH reporting / etc .

*Response: iHOST works with partner hospitals to develop new protocols for treatment of opioid withdrawal. It is not developing new treatments, rather it aims to reduce barriers to evidence-based treatments that are already recommended. The new protocols are agreed through the hospitals’ existing clinical governance structures and the hospitals remain responsible for the protocols and for identifying and investigating events that may be related to the safety of new protocols. An example of a relevant event might be a patient experiencing an opioid overdose after being administered OST in the hospital. Where such an event occurs and partner hospital assesses that the event was at least partly attributable to the new protocol, all partner hospitals together with the iHOST team will together review whether changes across all iHOST sites are necessary. We have clarified this procedure under the section “safety”.*
